# Capacitive-coupled Series Spoof Surface Plasmon Polaritons

**DOI:** 10.1038/srep24605

**Published:** 2016-04-19

**Authors:** Jia Yuan Yin, Jian Ren, Hao Chi Zhang, Qian Zhang, Tie Jun Cui

**Affiliations:** 1 State Key Laboratory of Millimeter Waves, Southeast University, Nanjing 210096, China; 2 Synergetic Innovation Center of Wireless Communication Technology, Southeast University, Nanjing, 210096, China; 3 State Key Laboratory of Millimeter Waves and Department of Electronic Engineering, City University of Hong Kong, Kowloon, Hong Kong SAR, China; 4 Cooperative Innovation Center of Terahertz Science, Chengdu 611731, China

## Abstract

A novel method to realize stopband within the operating frequency of spoof surface plasmon polaritons (SPPs) is presented. The stopband is introduced by a new kind of capacitive-coupled series spoof SPPs. Two conventional H-shaped unit cells are proposed to construct a new unit cell, and every two new unit cells are separated by a gap with certain distance, which is designed to implement capacitive coupling. The original surface impedance matching is disturbed by the capacitive coupling, leading to the stopband during the transmission of SPPs. The proposed method is verified by both numerical simulations and experiments, and the simulated and measured results have good agreements. It is shown that the proposed structure exhibits a stopband in 9–9.5 GHz while the band-pass feature maintains in 5–9 GHz and 9.5–11 GHz. In the passband, the reflection coefficient is less than −10 dB, and the transmission loss is around 3 dB; in the stopband, the reflection coefficient is −2 dB, and the transmission coefficient is less than −30 dB. The compact size, easy fabrication and good band-pass and band-stop features make the proposed structure a promising plasmonic device in SPP communication systems.

Surface plasmon polaritons (SPPs) appear when free electrons in metal are coupled with optical waves^1^,^2^. For a long time, SPPs have been considered to exist only in visible frequencies until Pendry and co-workers proposed a new structure to mimic the SPP characteristics at lower frequencies in 2004^3^,^4^. Since then, plenty of efforts have been made to construct plasmonic metamaterials to realize spoof SPPs in microwave and terahertz bands^5^,^6^,^7^,^8^,^9^,^10^,^11^,^12^,^13^,^14^. Subwavelength corrugated structures have been regarded as one of the most effective ways to propagate spoof SPPs, such as the perfectly conducting wire tailored by a periodic array of radial grooves on its surface^9^, corrugated V-shaped grooves^11^, corrugated metallic wedges^12^, and ultrathin planar corrugated metallic strips^13^, which are typical plasmonic metamaterials to support SPP-like modes. In the microwave and terahertz frequencies, another crucial problem is the efficient generation of SPPs, or the efficient conversion between the conventional spatial modes and SPP modes. For this purpose, several methods have been proposed to realize high-efficiency conversions from the conventional coplanar waveguide (CPW)^15^,^16^ and microstrip^17^,^18^ to the spoof SPP waveguide.

After a set of sophisticated theories of plasmonic metamaterials are gradually established and conceptually approach to maturity, the plasmonic functional devices have become hot topics to be investigated. Various kinds of wave splitters^19^,^20^, antennas^21^,^22^,^23^,^24^,^25^ and other devices have been reported in the microwave and terahertz bands. Meanwhile, SPP-based filters have spurred a long-held interest^16^,^26^,^27^,^28^,^29^,^30^. There are many approaches to realize filters with different characters. For instance, electrically resonant metamaterials have been used to produce tight coupling and mismatch of surface impedance, realizing the rejection of spoof SPPs^16^. Thus a kind of band-stop filter has been achieved. Combining the SPP transmission line with substrate integrated waveguide (SIW), another approach has been presented^29^ to realize the band-pass filters by using the low-pass feature of spoof SPP waveguide together with the high-pass feature of SIW. However, such two kinds of filters need additional structures to control the frequency-selective characteristics, making bulky volumes. In ref. 27, a novel way to realize band-pass filter was proposed by using a periodic subwavelength metallic Domino-block array. The transition bandwidth can be controlled by the geometry of the periodic structure, which is convenient except for the bulky dimension. Recently, an ultrathin planar frequency-selective SPP structure has been presented^26^, which is composed of two oppositely-oriented single-side corrugated metallic strips coupled to two double-side corrugated metallic strips. Although this design is based on the planar structure, it still has a relatively large volume with three rows of metallic strips.

In this work, we propose a capacitive-coupled series spoof SPP waveguide, in which every two H-shaped unit cells in the ultrathin corrugated metallic strip^15^ are regarded as a new unit cell in this particular design. Most importantly, every two new unit cells are separated by a gap with certain distance along the transmission direction, which can be equivalent to a capacitance. The capacitance among unit cells will make the original surface impedance become mismatched, bringing in a stopband in the original operating frequencies of spoof SPPs. The presented method has been verified by both numerical simulations and measurements. Compared with the existing SPP filters, the proposed structure is very compact without any additional components, is easy for fabrication, and has good performance of band-pass and band-stop characteristics, which are important to the plasmonic integrated circuits and communication systems.

## Results

**Figure 1:**
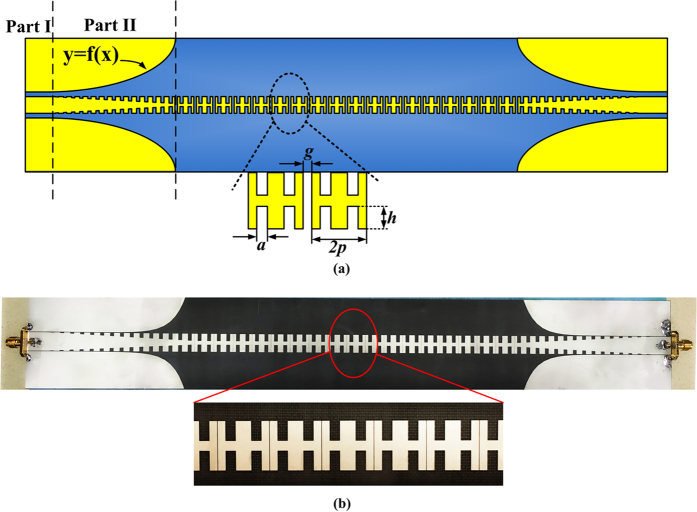
(**a**) Schematic picture of the proposed structure, in which the yellow part is metal (modeled as copper, a kind of lossy metal, the conductivity of which is 5.8 × 10^7^ S/m) and the blue part is dielectric substrate. (**b**) Prototype of the proposed structure.

As shown in Fig. 1(a), the proposed structure is designed on a 0.5 mm-thick substrate with permittivity 2.65 and loss tangent 0.003. The overall size is 349 × 51.1 mm^2^, with the thickness of the metal film is set as 0.018 mm. To achieve the broadband impedance matching, the CPW part (Part I) is designed with the width of central conductor as 10 mm and the gap between the central conductor and ground as 0.55 mm, to realize the 50-ohm impedance. The conversion part (Part II) from CPW to the SPP waveguide is similar to that in ref. 15, in which the gradient groove depth varies from zero to 4 mm, to reach the broadband momentum matching, and the optimized curve of flaring ground is described as an exponential function (*y* = *C*_*1*_*e*^*αx*^ + *C*_*2*_, *α* = 0.1, where *C*_*1*_ and *C*_*2*_ are determined by the length of the Part II and the width of the flaring ground) for impedance matching simultaneously. The overall length of Part II is 60 mm while the width of the flaring ground is set as 20 mm. As spoof SPPs appear along the transmission line after the conversion from spatial modes in CPW, the capacitive coupling should be designed. Every two H-shaped unit cells of the SPP waveguide is regarded as a new unit cell in this particular design. The dimensions of the original SPP unit cell are *p* = 5 mm, *a* = 2 mm, and *h* = 4 mm, denoting the period, width, and depth of grooves, respectively. Every two new unit cells are separated by a gap with certain distance of *g* = 0.1 mm after optimization, which can be equivalent to a capacitance. The capacitance added here will bring about a stopband within the operating frequencies of the original SPP waveguide due to the mismatch of the surface impedance.

**Figure 2:**
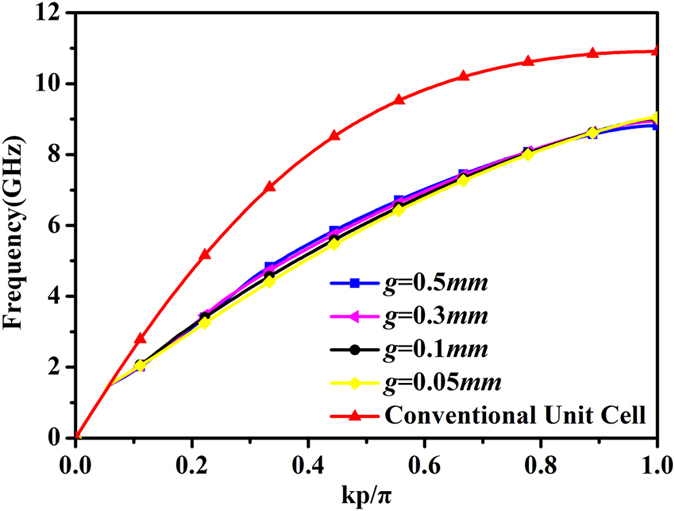
Dispersion curves of the new unit cell with different gaps and H-shaped unit cell in the original SPP waveguide. *p* is the period of the conventional unit cell as mentioned above and *k* is the wavenumber in the transmission line.

**Figure 3:**
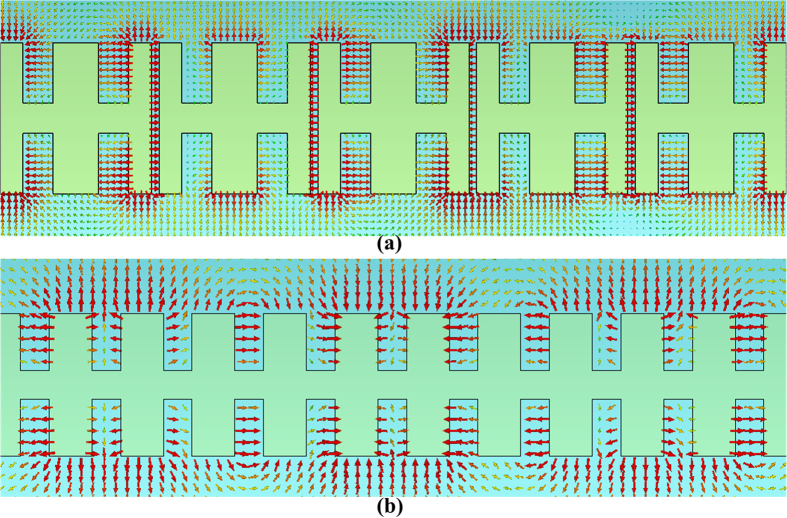
(**a**) The electric-field distribution of the proposed structure at 7 GHz, from which a resonance is observed within the gaps among new unit cells. (**b**) The electric-field distribution of traditional SPP transmission line at 7 GHz.

Figure 2 gives the dispersion curve of the new unit cell as well as the dispersion curve of the H-shaped unit cell in the original SPP waveguide. The simulations are completed by the commercial software, CST Microwave Studio. As mentioned above, there is a certain distance between the two new unit cells. Therefore the distance gap between new unit cells was also considered in simulations. As the comparison between different dispersion curves under different values of the gap is shown, it can be observed that the cutoff frequency of the new unit cell (around 9 GHz) is much lower than that of the H-shaped unit cell (11 GHz), indicating that there is a cutoff point at around 9 GHz when the energy propagates along the designed SPP waveguide. However, the propagation will not come to an end. Owing to the gap between the new unit cells, there is a resonance caused by the capacitance. The electric-field vector at 7 GHz given in Fig. 3(a) as an example illustrates the resonance clearly. Figure 3(b) shows the electric-field vector at the same frequency for comparison. With reference to the figure, we note that there is strong electric field existing within the gaps. Although the propagation along the SPP waveguide is cutoff, the energy can be propagated through the resonance. That is the reason why there may be one more passband after the stopband. On the other hand, the traditional SPP transmission line composed of H-shaped unit cells behaves as a low-pass filter. When the slots are introduced among the unit cells, it is equivalent to introducing a stopband during the initial operating band. That is to say, the initial passband is divided into two parts by the stopband, resulting in two passbands.

**Figure 4:**
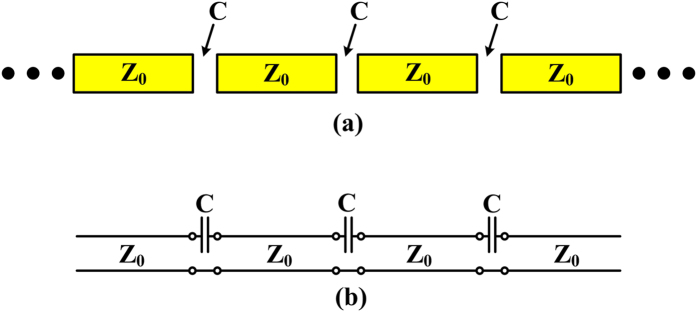
(**a**) Schematic of the proposed capacitive-coupled series spoof SPPs, in which the yellow part represents the spoof SPP waveguide. (**b**) The equivalent circuit model.

**Figure 5:**
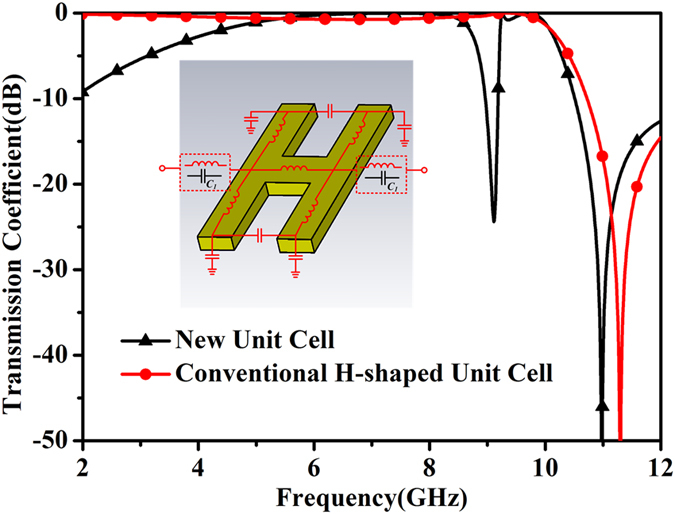
Simulated transmission coefficients of the two coupling cases, as well as the detailed circuit model.

In order to verify the above conjectures that there is another pass band above the cutoff frequency of the new unit cell, the propagation characteristic is analyzed using the microwave network theory. Firstly, as shown in the schematic of the proposed capacitive coupled series spoof SPPs in Fig. 4(a), the equivalent circuit model can be simplified to a series circuit shown in Fig. 4(b). Here, each unit cell can be modeled as a transmission line with a characteristic impedance of *Z*_*0*_ in series with a capacitor *C*_*1*_, which signifies the capacitance response caused by the slot. The detailed calculation of the characteristic impedance *Z*_*0*_ can be found in ref. 31 and the value of the series capacitor *C*_*1*_ can be quantitative analyzed by some complicated co-simulation between CST Microwave Studio and Advanced Design System (ADS). Then we take one of the segments for calculation, and the transmission matrix can be deduced as:
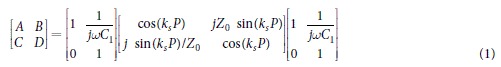
where *k*_*s*_ is the wavenumber of spoof SPPs, P is the period of the unit cell. For clearance, the detailed circuit model is shown in Fig. 5. The dashed box means there are two kinds of coupled modes in the proposed transmission line, namely inductive coupling and capacitive coupling. When the H-shaped unit cell is set next to each other with no distance, it can be considered that there is an inductance, while the H-shaped unit cell is separated to the other with a certain distance, a capacitance can be assumed here. The capacitance *C*_*1*_ in Fig. 5 is the same one with that in the equation above. According to the detailed model, the transmission coefficients of two kinds of unit cells are simulated using ADS. The transmission coefficients of the two coupling cases are also shown in Fig. 5. With reference to the figure, it can be seen that for the inductive coupling, responding to the conventional SPP transmission lines, low-pass characteristic can be observed. This is consistent with the typical transmission characteristic of the SPP transmission lines in the open literatures. When the capacitive coupling is introduced to the transmission line, a stop band can be observed clearly. By change the width of the slot, the coupling capacitance can be changed, resulting in the frequency shifting of the stopband. This will be discussed latter. In brief, the simulated results can be applied to prove the validity of the proposed circuit model and the deduced equations based on it.

**Figure 6:**
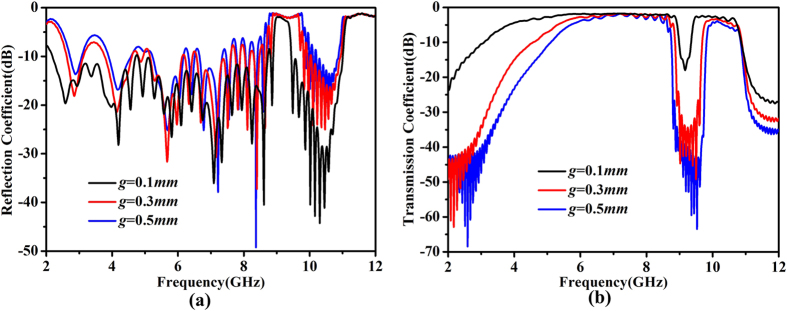
Simulated S-parameters with different values of gap distance *g* between two unit cells. (**a**) Reflection coefficients. (**b**) Transmission coefficients.

To get a better understanding on the influence of the capacitance between new unit cells, the reflection and transmission coefficients are simulated under different gap distances. With reference to Fig. 6, it can be observed that when the gap distance between new unit cells increases, the frequency range of stopband becomes wider, owing to the larger mismatch of the surface impedance. This is consistent to the predictions before. It is worth noting that the cutoff frequency of the first passband agrees well with the dispersion curve in Fig. 2. However the big mismatch will also lead to the bad performance of the structure. We note that the reflection coefficient is above −10 dB during the passband, and the transmission loss becomes larger in both passbands for wider gap at the same time, especially in the second passband. On the basis of the definition of the bandstop filter, the energy can be transmitted under majority frequencies but reflected under some appoint frequencies, so here the performances of passband and stopband are both taken into account when measure the performance of the whole structure. According to the general relationship between the performance and the gap distance among unit cells, a narrower stopband and better performance in passband can be realized by a smaller distance than 0.1 mm. Nevertheless, due to the processing condition, the gap cannot be smaller than 0.1 mm, and hence we set the final gap as 0.1 mm. Other ways to increase the capacitance between unit cells (e.g. the interdigital capacitive structure) can also be considered in the future work.

**Figure 7:**
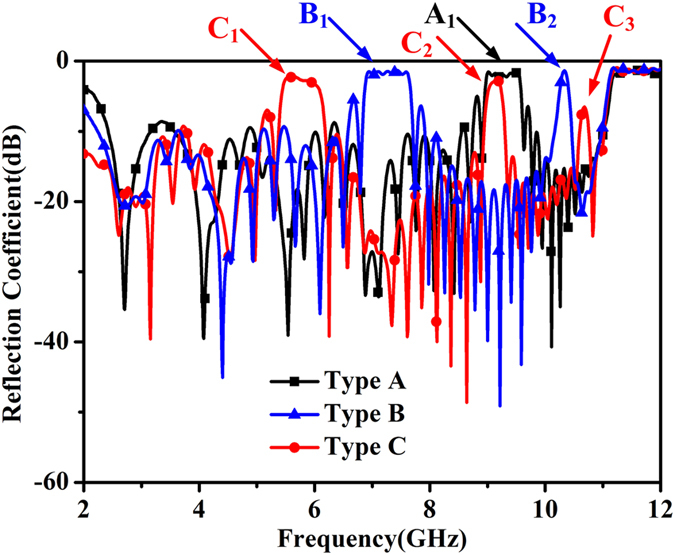
Simulated reflection coefficients of different kinds of new unit cell. Type A is the initial design in this paper. Three traditional unit cells compose the unit cell of Type B, while four traditional unit cells compose the unit cell of Type C. The letters with subscript signify the stopband of relevant type.

After the value of the gap is determined, another parameter should be studied for further understanding. In order to find the effect of the location of the gap, different kinds of new unit cells are used to make simulation. The initial unit cell of this paper is composed of two conventional unit cells. This kind of design is called Type A here for simplification. For comparison, if three conventional unit cells compose of a new unit cell, it is called Type B. And Type C is for the unit cell composed of four conventional unit cells. The simulated reflection coefficients of these three kinds of design are given in Fig. 7. A_1_ indicates the only stopband of Type A, while B_1_ and B_2_ are the two stopbands of Type B. Similarly, C_1_, C_2_ and C_3_ are the three stopbands of Type C. From the simulated results, it can be observed that the number of the stopbands is related to the number of the conventional unit cells. The appearance of each stopband can be predicted in a similar way through the analysis above.

**Figure 8:**
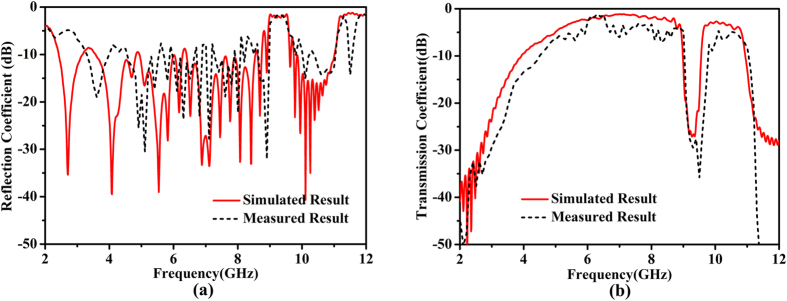
(**a**) Measured reflection coefficients compared with the simulated results. (**b**) Measured transmission coefficients compared with the simulated results.

In experimental demonstrations, the proposed structure was fabricated, as shown in Fig. 1(b). All materials in the experiment are the same as those in the simulation. The spectrum responses in Fig. 8(a,b) present a desirable consistency between simulated and measured results, except for a slight deviation in the second passband, which is resulted from the fabrication error and matching error. Meanwhile a larger transmission loss is observed in the experiment, which is mainly due to the ignorance of dielectric loss and metal loss in the simulations. From the measured results, it can be seen that the reflection coefficient is less than −10 dB and the transmission loss is around 3 dB in the passband; while the reflection coefficient is −2 dB and the transmission coefficient is less than −30 dB in the stopband. The good performance suggests that the capacitive coupling to bring about stopband completes the mission successfully.

**Figure 9:**
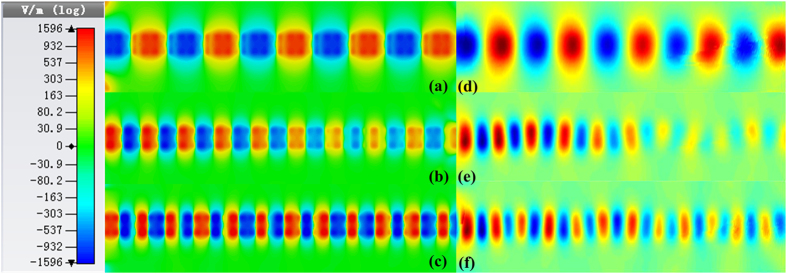
Near-electric-field distributions of the proposed structure. (**a**) Simulated result at 6 GHz. (**b**) Simulated result at 9 GHz. (**c**) Simulated result at 10 GHz. (**d**) Measured result at 6 GHz. (**e**) Measured result at 9 GHz. (**f**) Measured result at 10 GHz.

In order to demonstrate the transmission character visually, the near-electric-field distributions of the proposed structure are given in Fig. 9, in which (a)–(c) are the simulated near fields at different frequencies on an observation plane that is 2 mm above the structure, while (d)–(f) illustrate the corresponding measured results. The frequency points are chosen as 6, 9, and 10 GHz, representing the first passband, stopband, and the second passband, respectively. Due to the limitation of measured length, only the transmission part has been measured and the near-field distributions of the conversion part from CPW to SPP waveguide are not given in Fig. 9. From the near-field distributions, one can clearly see that the energy cannot propagate in the stopband, while the energy propagates steadily in the passband, in which we observe good agreements between the simulated and measured results. The good measurement results imply that the proposed structure is a promising device for the SPP integrated circuits and systems.

## Discussion

In summary, a novel method was proposed to realize stopband during the operating frequencies of conventional spoof SPPs by introducing capacitive-coupled series structures. The new unit cell of this particular design is composed of two conventional H-shaped unit cells, and every two new unit cells are separated by a gap distance, which is designed to implement capacitive coupling. The original surface impedance matching is then disturbed by the capacitive coupling, leading to the stopband during the transmission procedure. The proposed method was verified by both simulation and measurement results, which have good agreements. The proposed structure exhibits a stopband in 9–9.5 GHz; while the band-pass characteristics maintain in 5–9 GHz and 9.5–11 GHz. In the passband, the reflection coefficient is less than −10 dB and the transmission loss is around 3 dB; while in the stopband the reflection coefficient is −2 dB and the transmission coefficient is less than −30 dB. Without using any additional structures, the proposed device is compact, easy for fabrication and integration, and has good performance of band-pass and band-stop characteristics. Such advantages are very important to the SPP integrated circuits and systems.

## Methods

Numerical simulations are performed by the commercial software, CST Microwave Studio. The experimental structure is fabricated using a 0.5-*mm* thin dielectric film with dielectric constant 3 and tangent loss 0.03, respectively. The thickness of metal (copper, a kind of lossy metal, the conductivity of which is 5.8 × 10^7^ S/m) film is 0.018 *mm*. We use Agilent Vector Network Analyzer to measure the *S* parameters (i.e., the reflection coefficients *S*_*11*_ and transmission coefficients *S*_*21*_) of the fabricated sample. The near electric-filed distributions are measured by a home-made near-field scanning system, in which the probe is set as 2 *mm* above the fabricated sample.

## Additional Information

**How to cite this article**: Yin, J. Y. *et al*. Capacitive-coupled Series Spoof Surface Plasmon Polaritons. *Sci. Rep.* **6**, 24605; doi: 10.1038/srep24605 (2016).
